# Optimization of the Recovery of Secondary Metabolites from Defatted *Brassica carinata* Meal and Its Effects on the Extractability and Functional Properties of Proteins

**DOI:** 10.3390/foods11030429

**Published:** 2022-02-01

**Authors:** V. P. Thinh Nguyen, Jon D. Stewart, Florent Allais, Irina Ioannou

**Affiliations:** 1 URD Agro-Biotechnologies Industrielles (ABI), CEBB (Centre Européen de Biotechnologie et de Bioéconomie), AgroParisTech, 51110 Pomacle, France; vpthinh.nguyen@icloud.com (V.P.T.N.); florent.allais@agroparistech.fr (F.A.); 2 Department of Chemistry, University of Florida, Gainesville, FL 32611, USA; jds2@chem.ufl.edu

**Keywords:** phenolic compounds, glucosinolates, Response Surface Methodology, proteins, *Brassica carinata*

## Abstract

The sustainable extraction of secondary metabolites from Brassica agro-industrial by-products often involves the use of high concentrations of ethanol, and/or high temperatures, which tends to decrease the efficiency of protein extraction (yield, profile, etc.). To understand the limits of the combination of these two extraction processes, aqueous ethanol extraction of secondary metabolites (e.g., phenolic compounds and glucosinolates) from *Brassica carinata* defatted meal was optimized using Response Surface Methodology. The validated models predicted that aqueous ethanol extraction of defatted Carinata meal, with a low aqueous EtOH concentration (22% EtOH) at moderate *T_e_* (50 °C), enables the efficient recovery of secondary metabolites (sinapine = 9.12 ± 0.05 mg/g_DM_, sinigrin = 86.54 ± 3.18 µmol/g_DM_) while maintaining good protein extractability (59.8 ± 2.1%) from successive alkaline extractions. The evaluation of functional properties of the resulting protein isolates revealed that aqueous extraction, under optimized conditions, improves foaming activity while preserving emulsion ability.

## 1. Introduction

Historically cultivated in the Ethiopian highlands and adjoining areas of East African and Mediterranean regions for food purposes, *Brassica carinata* (referred as Carinata) is now considered a nonfood oilseed crop in the United States due to its high erucic acid-rich oil content, which is suitable for aviation biofuel production [1]. Cultivating Carinata in the southeast regions of the USA is believed to be economically and ecologically beneficial for local farmers, by improving soil nutrient and moisture conservation during winter seasons (Southeastern Partnership Advanced Renewable from Carinata [2]. Although the main purpose of such cultivations is to support bio-fuel production, the residual meal still contains valuable components, such as proteins and secondary metabolites [3,4].

The defatted Carinata meal obtained after oil extraction could be an important source of proteins. Indeed, the nutritional value of Carinata is similar to that of rapeseed (*B. napus*), which is considered to be well-balanced for human nutrition [5,6,7]. Moreover, the nutritional value of Carinata is comparable to that of other plant-based proteins (soybean, sunflower), and even certain animal-based proteins (casein) [3]. It should be noted that these proteins also have valuable functional properties [8,9] for many applications (e.g., encapsulation of bioactive compounds, drug delivery) [10,11]. The secondary metabolites of Carinata meal are mainly phytic acid, phenolic compounds (PCs), and glucosinolates (GSLs) [7,12,13]. Although the therapeutic benefits of these co-product molecules have often been mentioned (e.g., antioxidant, antibacterial, anticancer) [7,14,15,16], they also exhibit anti-nutritional activities at high concentrations, and thereby decrease the nutritional value of meal or protein extracts [7,17]. An additional separation step is, therefore, required to remove secondary metabolites from protein extracts. Such a separation process must have a high separation efficiency, without altering the extractability or functional properties of the proteins.

Sustainable and environmentally friendly recovery of bioactive molecules from biomass is attracting ever-increasing attention [18]. The recovery of these metabolites from agro-industrial wastes using aqueous alcohol extraction has become popular [12,19,20,21,22]. Although aqueous methanol has traditionally been used to recover phenolic compounds from biomass [23,24], aqueous ethanol (AE) has become more attractive, thanks to its low toxicity [20,22]. Nevertheless, such an extraction process often involves the use of alcohol at high concentrations and/or the use of high temperatures, which can partially alter the solubility and functional properties of proteins, due to structural changes [25,26]. To the best of our knowledge, very few studies have focused on the extractability and functional properties of the proteins contained in meal after AE extraction. Indeed, only one study on rapeseed meal has been reported [27], in which the effect of AE extraction under defined conditions (70% EtOH/ultrapure water, room temperature) resulted in an 11% decrease in protein extractability.

In this article, we carry out a 2-step fractionation of Carinata meal: an AE extraction to recover the secondary metabolites, followed by an alkaline extraction to obtain protein extracts. The objective is to study the effects of AE extraction of secondary metabolites on protein extractability by observing the protein content and polypeptide profile of the extracts. For this, the AE extraction process was optimized using RSM, to maximize either the content of PCs, the content of GSLs, or the content of proteins. Secondly, different optimal operating conditions were compared, and a compromise was found which allowed the extraction of large amounts of PCs and GSLs while maintaining good protein extractability. The effect of these conditions was also evaluated on the functional properties of the residual proteins.

## 2. Materials and Methods

### 2.1. Chemicals

Sinigrin hydrate and sinapic acid, used as external standards, were purchased from Sigma-Aldrich. Pure synthetic sinapine chloride was synthesized in our laboratory and used as the standard for HPLC analysis [22,28]. The ethanol solution used for extraction was purchased from ThermoFisher. LC-MS grade formic acid and acetonitrile, for HPLC analysis, were purchased from ThermoFisher. MilliQ water was produced by Milli-Q Direct 8 (Merck Millipore, MA, USA).

### 2.2. Carinata Meal Samples and Their Characterization

Hexane-solvent extracted Carinata meal was provided by Dr. DiLorenzo (IFAS, University of Florida, FL, USA). The meal was ground, passed through a 30-mesh screen (500 microns), and stored at −20 °C until use. The water content of the meal was determined to be 88.8 ± 0.4 mg/g of Carinata meal using a standard moisture analyzer; MB35-OHAUS™ (OHAUS, New Jersey, NJ, USA). Ash and protein contents were determined using AOAC approved methods [29]. Soluble carbohydrate and phytic acid contents were determined using the protocol described by Dubois et al. and Reichwald and Hatzack, respectively [30,31].

### 2.3. Fractionation Process of Carinata Meal

**Figure 1:**
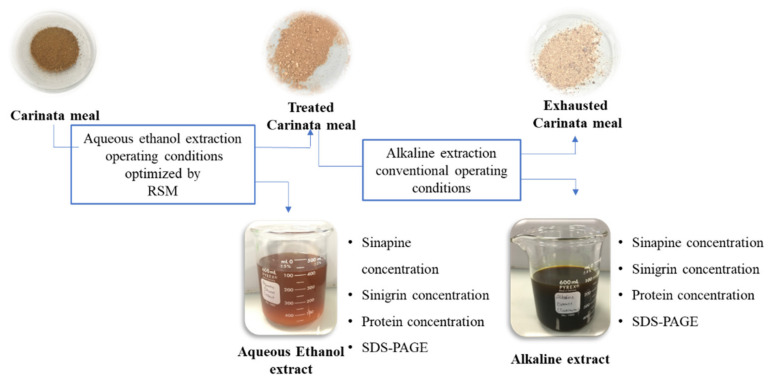
Fractionation of Carinata meal.

The fractionation process involves two successive extractions, as shown in Figure 1.

Five grams of defatted *B. carinata* meal were extracted with 50 mL of a mixture of EtOH/MilliQ water for 1 h at 300 rpm. The EtOH concentration and extraction temperature varied according to the design of the experiment, as presented below. The slurry was centrifuged for 20 min at 4000 rpm and 25 °C using a refrigerated benchtop centrifuge Allegra X15-R (Beckman Coulter, Pasadena, CA, USA). After centrifugation, the supernatant (henceforth referred as the AE extract) and residual meal (pellet) were recovered. The AE extract was evaporated under vacuum to remove EtOH. The concentrated liquid was freeze-dried, weighed, and stored at room temperature. The contents of sinapine and sinigrin in the AE extracts were determined, along with the nitrogen solubility and the polypeptide profile. The residual meal was air-dried overnight (referred as treated Carinata meal), and then stored at 4 °C for a maximum of 2 days prior to alkaline extraction.

Alkaline extraction was performed following the published procedure, with slight modifications [3]. In brief, treated Carinata meal was extracted with 50 mL of 0.1 M KOH at 300 rpm for 30 min. A pH value 12 was recorded using a pH meter SevenCompact pH/Ion meter S220 (Mettler Toledo, OH, USA). The slurry was centrifuged for 20 min at 4000 rpm and 25 °C. After centrifugation, the supernatant (protein extract) and pellet (exhausted meal) were recovered. The protein extract was freeze-dried, weighed, and stored at room temperature. The same analysis used previously for the AE extracts was also carried out for the protein extracts. The control was represented by protein extract obtained from the original Carinata meal by alkaline extraction (with no prior AE step).

### 2.4. Determination of Phenolic Compound and Glucosinolate Contents

HPLC quantification of PCs was performed as described elsewhere [22] on a UHPLC-DAD system (Ultimate 3000, Dionex, ThermoFisher, MA, USA) equipped with a quadratic pump, auto sampler, column furnace, and diode array detector. Data were analyzed with Chromeleon software (Version 6.8, Thermofisher, MA, USA). Main PCs, including sinapine and sinapic acid, were identified by comparing their relative retention time to a standard. Synthetic sinapine chloride was used in the preparation of the calibration curve.

The GSL content was determined following procedure described by Grosser and van Dam, with some modifications [32]. Samples were diluted ten times prior to the desulfation step. One milliliter of sample was loaded onto a glass pipette, preconditioned with 0.5 mL of Sephadex A25 0.1 mg/mL in MilliQ-water. A total of 2 × 1 mL of MilliQ-water was used for washing. The column was next washed with 2 × 1 mL of acetate buffer (40 mM, pH 5.5). Twenty microliters of cleaned-up Helix pomatia sulfatase solution (S9626, Sigma-Aldrich) was added onto the column, followed by flushing with 50 µL of acetate buffer. The column was incubated at room temperature prior to eluting with 2 × 1 mL of MilliQ-water. The eluted fraction was then analyzed by UHPLC.

### 2.5. Determination of Protein Content of AE Extracts

The protein content of AE extracts was calculated based on the nitrogen solubility, according to the AOAC approved method [29], and is shown in Equation (1):

(1) $$C_{protein}=\frac{\%N\times 6.25\times m_{\mathrm{lyoph}\;\mathrm{extract}}}{m_{\mathrm{DM}}},$$

where %N is the nitrogen content of freeze-fried extracts, determined by combustion following the Dumas method. M_lyoph extract_ is the mass of freeze-dried supernatant (g), and m_DM_ is the mass of dry matter in Carinata meal (g).

The nitrogen content (%N) of freeze-dried AE and protein extracts was determined using the Dumas method, performed by UMR 614 Institut National de la Recherche Agronomique-Université de Reims Champagne-Ardennes (INRA-URCA) commercial services.

### 2.6. Analysis of Polypeptide Profile

The polypeptide profiles of AE and alkaline extracts were analyzed using electrophoresis. Sodium dodecyl sulfate polyacrylamide gel electrophoresis (SDS-PAGE) was carried out following standard procedure under non-reducing conditions. In brief, around 10 mg of freeze-dried supernatant was dissolved in 1 mL of MilliQ-water, with vortex mixing to resuspend the solid. Fifteen microliters of this solution were mixed with 5 µL of 4X Laemmli Sample Buffer (Biorad, Hercules, CA, USA). The mixture was boiled for 15 min and cooled at room temperature for 30 min. Twenty microliter aliquots were then loaded onto the precast gradient gels MiniProtein TGX 4–15% Strain Free (Biorad, Hercules, CA, USA), along with 15 µL of standard proteins SpectraTM Multicolor Broadrange (Thermo Fisher, Waltham, MA, USA). Separation of samples was performed for 1 h at 20 mA per gel.

### 2.7. Optimization of the AE Extraction of Secondary Metabolites

A D-optimal design was used to optimize the operating conditions of the AE extraction of secondary metabolites, with a total of 13 experiments and a triplicate at the central point (Table 1). The independent variables used in this optimization were the concentration of ethanol (%EtOH, X_1_) and the extraction temperature (T_e_, X_2_). The significant contributions of %EtOH and T_e_ to the recovery yield of secondary metabolites were recently reported [20,22]. As a result, %EtOH and T_e_ were varied to four different levels (25, 45, 70 and 90%) and three different levels (25, 50 and 75 °C), respectively. The limit was set at 75 °C to avoid loss of solvent by evaporation. Other solid–liquid extraction parameters, including extraction time, solvent–solid ratio, and extracting materials’ particle size, remained constant, as these were thought to be non-significant in the context of our study.

Three responses were optimized: the phenolic compound content (Y_1_), the sinigrin content (Y_2_), and the extractability index of proteins (Y_3_) of the AE extracts.

Under some conditions, sinapine can be hydrolyzed in sinapic acid, these two molecules being the two main phenolic compounds in Carinata meal. Thus, the PCs determined herein include both the sinapine and sinapic acid contributions. Y_1_ is expressed according to the dry matter and calculated as shown in Equation (2):

(2) $$Y_{1}=\frac{\left(C_{\mathrm{sinapine}}+C_{\mathrm{sinapic}\;\mathrm{acid}}\right)\times {\;m}_{\mathrm{extract}}}{d\;\times {\;m}_{\mathrm{DM}}},$$

where C_sinapine_ is the concentration of sinapine in AE extract (in mg/mL), C_sinapic acid_ the concentration of sinapic acid in AE extract (mg/mL), m_extract_ the mass of extract (g), d the volumetric mass density (g/mL), and m_DM_ the mass of dry matter in Carinata meal (g).

The GSL content of AE extracts (Y_2_) is expressed according to the dry matter and calculated as shown in Equation (3):

(3) $$Y_{2}=\frac{C_{\mathrm{GSL}}\times \left(m_{\mathrm{extract}}\right)}{\;d\;\times {\;m}_{\mathrm{DM}}},$$

where C_GSL_ is the concentration of GSL (as sinigrin) in AE extract (µmol/mL), m_extract_ is the mass of the extract (g), d is the volumetric mass density (g/mL), and m_DM_ the mass of dry matter in Carinata meal (g).

AE extraction mainly impacts their extractability (Y_3_) of proteins in this optimization study. The protein content of the different extracts was calculated according to Equation (1). By taking the protein content of the control (alkaline extracts from untreated Carinata meal) as the maximum content that can be extracted, we can define an extractability index, as described in Equation (4):

(4) $$Y_{3}=\frac{C_{\mathrm{protein}}\times m_{\mathrm{extract}}}{C_{\mathrm{protein}\;\mathrm{control}}\times m_{\mathrm{control}}}\times 100\%,$$

where C_protein_ is the protein content of alkaline extract from residual meal upon AE extraction (mg/g_DM_), m_extract_ is the mass of the extract (g), and C_protein control_ is the protein content of the alkaline extract from the control Carinata meal (mg/g_DM_).

The experimental data were fitted using a second-order polynomial equation:

(5) $$Y_{q}=\beta_{0}+\sum_{i=1}^{2}\beta_{i}X_{i}+\sum_{i=1}^{2}\beta_{\mathrm{ii}}X_{i}^{2}+\sum \sum_{i<j=1}^{2}\beta_{\mathrm{ij}}X_{i}X_{j}+\varepsilon_{\mathrm{residues}\;q},$$

Yq is the different responses (q = 1–3); β0, βi, βij, and βii are the regression coefficients of the mean, linear, interaction, and quadratic terms, respectively; Xi and Xj are the independent variables; and ε_residues q_ is the difference between the observed and the predicted value.

Experimental design and analysis were performed using the software MODDE v.12.0 (Satorius, Gottingen, Germany). An analysis of variance (ANOVA) with 95% confidence level was carried out for each response to test the model significance and adequacy. The model coefficients were considered as significant when the Student’s *t*-tests of significatively give a *p*-value inferior to 0.05. Surface responses were plotted for each response in order to observe the effects of the different terms of the model.

### 2.8. Determination of Optimal Operating Conditions

MODDE—a Nelder–Mead simplex method-based software optimizer tool—was used to find the optimal operating condition for the recovery of secondary metabolites.

To determine those maximizing the recovery of secondary metabolites and protein extractability, Microsoft Excel (Version 16.43) was used. Indeed, the Y_1_, Y_2_, and Y_3_ responses vary antagonistically, and the MODDE optimizer tool was defeated. We plotted graphs in which Y_1_, Y_2_, and Y_3_ values were calculated using prediction models according to the %EtOH for a fixed extraction temperature. Thus, six graphs were plotted for extraction temperatures ranging from 25 to 50 °C in five-degree steps. T_e_ was limited to 50 °C to avoid the dissociation of certain proteins, such as cruciferin [33]. The optimum zone, corresponding to the meeting point of the three models for which the three responses were the highest, was determined for each graph.

### 2.9. Functional Properties

#### 2.9.1. Preparation of Acid-Precipitated Isoelectric Protein Isolates

Aluko and McIntosh’s procedure was used to produce acid-precipitated isoelectric protein isolate for functional property analyses [34]. In brief, Carinata meal was extracted with 10 volumes of 0.1 M NaOH for 30 min at 300 rpm and room temperature. The slurry was then centrifuged for 20 min at 4000 rpm and 25 °C. The supernatant was then separated from the residue and gradually adjusted to pH 4 using 1 M HCl. The precipitate was then recovered by centrifugation, washed twice with 10 volumes of MilliQ-water, freeze-dried, weighed, and stored at −20 °C until further analyses.

#### 2.9.2. Emulsifying Activity Index

The emulsifying activity index (EAI) was determined following the procedure described by Aluko and McIntosh [34]. Protein solutions (1% *w/v*) were prepared in 0.01 M phosphate buffer (pH 7.0), and 5 mL of the latter was added to 1 mL of pure commercial canola oil. The protein solution and oil phase were homogenized at 16,000 rpm for 1 min using a T25 digital Ultra-Turrax (IKA-Works, Inc., Cincinnati, OH, USA). Ten microliters of the emulsion was immediately diluted to 5 mL with 0.1% (*w/v*) SDS solution (0.1 g/100 mL), and the absorbance at 500 nm was measured using the 0.1% SDS solution as a blank. The EAI (m^2^/g) was calculated as described in Equation (6), according to the Pearce and Kinsella method [35]. The emulsions were allowed to stand at room temperature for 30 min, and the EAI was determined and expressed as a percentage of the initial EAI to obtain emulsion stability (ES).

(6) $$\mathrm{EAI}=\frac{2\times 2.303\times A\times N}{C\times \varphi \times 10000},\;$$

where A is the absorbance recorded at 500 nm, N is the dilution factor (×500), C the protein concentration before the formation of the emulsion (g/mL), and φ, the volume fraction of the oil (mL).

#### 2.9.3. Foam Expansion (FE) and Foam Stability (FS)

FE was determined according to the protocol described by Aluko and McIntosh [34]. Ten milliliters of protein solution (1% *w/v*), prepared in 0.01 M sodium phosphate buffer (pH 7.0) was homogenized at 16,000 rpm for 30 s. The volume of foam obtained was expressed as a percentage of the initial volume of protein solution. FS was determined by measuring the volume of foam that remained after standing at room temperature for 30 min, expressed as a percentage of the initial foam volume.

### 2.10. Statistical Analysis

Each analysis was performed in duplicate. Results correspond to the mean associated to its standard deviation. Student’s *t*-test, ANOVA, and post-hoc analysis using Tukey’s test were carried out using RStudio (Version 3.6.1, RStudio Inc., Boston, MA, USA).

## 3. Results

Ash and protein contents were determined to be 72.0 ± 0.2 mg/g of dry matter (mg/g_Dry Matter (DM)_) and 499.1 ± 1.7 mg/g_DM_, respectively. Soluble carbohydrates corresponded to 74.7 ± 3.7 mg/g_DM,_ and the content of phytic acid was 7.6 ± 0.4 mg/g_DM_ using the colorimetric method. The remaining residue was composed of fibers and secondary metabolites, including PCs and GSLs. These chemical compositions are similar to those of defatted Carinata meal reported by other studies [3,36].

In a successful double valorization of Carinata meal, the extraction of secondary metabolites, carried out in the first step, must have a slight impact on the extractability and functional properties of the proteins obtained in the second extraction. In this context, the extraction process of PCs and GSLs was optimized, and the impact of the optimal operating conditions on proteins was studied. The AE extraction was performed prior to the alkaline extraction to avoid the amalgam extraction of proteins along with secondary metabolites, which would require a more complicated downstream separation are reported in Figures S1 and S2 (Supplementary Materials).

### 3.1. Optimization of Aqueous Ethanol Extraction using Response Surface Methodology

A D-optimal design was used to optimize the operating conditions for AE extraction of Carinata defatted meal based on the secondary metabolite contents. Two independent variables—%EtOH with four levels (25, 45, 70, and 90%) and T_e_ with three levels (25, 50, and 75 °C)—were selected as factors. The responses upon optimization were Y_1_ the PC content, Y_2_ the GSL content, and Y_3_ the extractability index of proteins.

#### 3.1.1. Model Adequacy

The D-optimal design generated by optimizer software resulted in 13 extractions. Multiple regression equations using polynomial second order were applied to afford the responses. Analysis of variance (ANOVA) was then conducted to determine the regression coefficients of each model. Table 2 summarizes the statistical parameters obtained in this study.

Coefficients were considered significant for *p* < 0.05. Non-significant coefficients were removed to obtain reduced models when the predictive capacity of the model was not decreased. The coefficients of determination of the three prediction models indicated good model accuracy (R^2^ > 0.8). The adjusted determination coefficients furthermore confirmed the adequacy of the established models (R^2^ adjusted > 0.75). The orthogonality of all models was determined to be satisfactory, as the condition numbers were inferior to 10. With good reproducibility values (>0.8), the relationships between the variables and the responses were well depicted by these models.

#### 3.1.2. Phenolic Compound and Sinigrin Contents in Aqueous Ethanol Extracts (Y_1_ and Y_2_)

**Figure 2:**
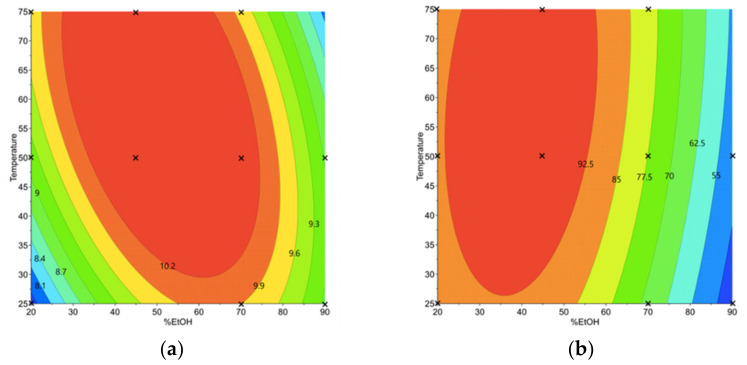
Contour plots for the prediction of phenolic compound (mg/g_DM_) (**a**) and glucosinolate contents (µmol/g_DM_) (**b**). The crosses indicate data points that were fitted for plot building.

Variations of $Y_{1}$ and $Y_{2}$ were found among the experiments of the D-optimal design. Y_1_ ranged from 7.26 to 11.33 mg/g_DM_ (Table S1), whereas Y_2_ ranged from 32.58 to 94.01 µmol/g_DM_ (Table S2). Model equations are given in Supplementary Materials. Response surfaces were plotted to see the effect of the different variables on the responses (Figure 2).

According to Table 2, the most significant terms in the model equation providing Y_1_ are the quadratic term for the ethanol content (%EtOH×%EtOH) and the interaction between the ethanol concentration and temperature (%EtOH× T_e_), which have a negative impact on the recovery of phenolic compounds (Figure 2a). These results are consistent with other studies [37,38]. Reungoat et al. showed that %EtOH×%EtOH leads to a negative effect on AE extraction of sinapine from mustard bran (−3 mg/gDM) [22]. In contrast to other studies, the quadratic term for T_e_ in this study was unsignificant. The use of different types of biomasses (defatted meal instead of bran) for the AE extraction was suggested to account for the differences observed [22,38]. The prediction model showed that the highest content of phenolic compounds (over 10.2 mg/gDM) was reached for a %EtOH varying from 40 to 70%. In contrast, the recovery was significantly decreased for an extraction carried out with a %EtOH less than 40% or greater 70%, as depicted in Figure 2.

According to Figure 2b, a negative effect of the quadratic term of ethanol concentration (%EtOH×%EtOH) on sinigrin recovery is observed, whereas the other terms related to T_e_ do not significantly contribute to the recovery of this metabolite. These results were expected, as a conventional sinigrin recovery process was used. Indeed, the latter being very hydrophilic due to the ionized sulfate and hydrophilic thioglucose moieties, a solid–liquid extraction with an alcohol/water mixture is appropriate [32,39]. However, since GSLs are highly hydrophilic, their content in AE extracts appears to be more sensitive to the variation of %EtOH than PCs. More than half of the amount of GSLs is reduced by increasing the %EtOH from 40% to 90%.

#### 3.1.3. Determination of the Optimal Conditions of Aqueous Ethanol Extraction and Validation of the Prediction Models

The results suggested that, through the AE extraction, PCs and GSLs can be simultaneously and optimally recovered from defatted Carinata meal. The optimal conditions, suggested by the MODDE software based on the previously established models, are %EtOH = 47% and T_e_ = 62 °C. Under these conditions, the contents of PCs and GSLs, predicted by the models, were determined to be 11.74 ± 1.26 mg/g_DM_ and 103.6 ± 16.2 µmol/g_DM_, respectively. These values remain statistically similar (Table S3) to the values observed (10.87 ± 0.54 mg/g_DM_ and 98.9 ± 4.9 µmol/g_DM_ for the PCs and GSLs, respectively), and thus confirmed the validity of the prediction models for Y_1_ and Y_2_.

### 3.2. Effects of Aqueous Ethanol Extraction on Carinata Proteins

The previous D-optimal design was also used to optimize Y_3_, the extractability index of the proteins in AE extracts.

#### 3.2.1. Extractability of Proteins Resided in Carinata Treated Meal (Y_3_)

Considering the characteristics of the proteins, their extractability can be influenced by the processes used prior their extraction [39]. Thus, the effect of the AE extraction process of secondary metabolites on the extractability of proteins (Y_3_) was studied and quantified using RSM. Variations of $Y_{3}$ were found among the experiments of D-optimal design, and ranged from 41.3 to 54.5 % (Table S4).

**Figure 3:**
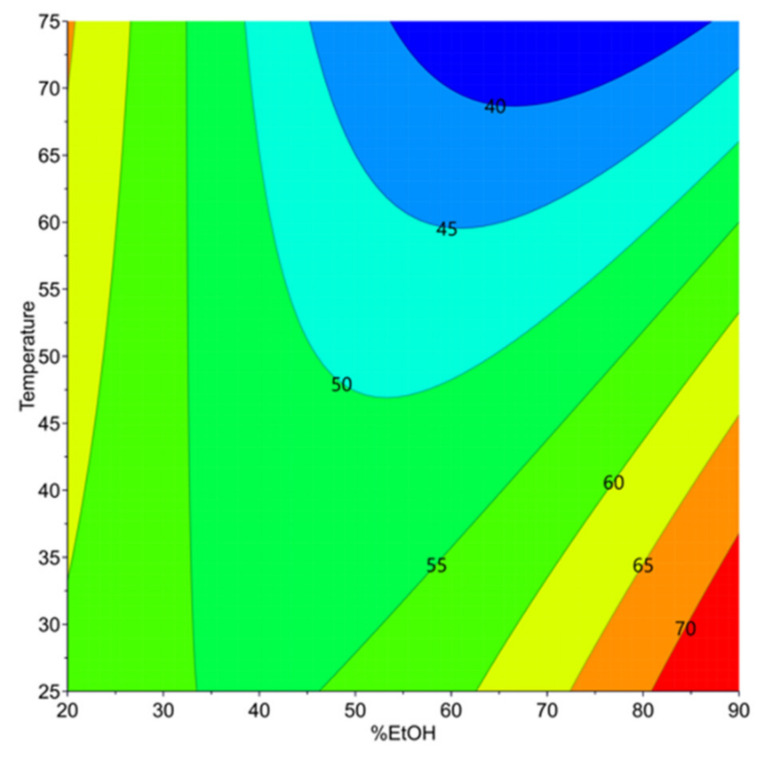
Contour plot for the prediction of extractability index (%) of proteins. Dashed red triangle indicates optimal zone of protein extractability index.

Optimal zones to obtain a high extractability index of proteins are shown in Figure 3.

According to Table 2, the most significant terms in the model equation providing Y_3_ are the quadratic term for the ethanol concentration (%EtOH×%EtOH), which has a positive effect on the protein extractability and the extraction temperature T_e_, and the interaction term (T_e_×%EtOH), which has a negative impact (Figure 3).

The prior AE extraction of secondary metabolites decreases the extractability of proteins during alkaline extraction, regardless of the operating conditions. The prediction model indicates a sharp decrease in protein extractability (Y_3_) when moderate %EtOH (40–70%) and T_e_ above 45 °C are applied. This is due to the high protein recovery with secondary metabolites during AE extraction, resulting in lower protein availability in the treated meal during alkaline extraction.

The prediction model also indicates that a high extractability of the proteins is retained when AE extraction of secondary metabolites is performed with a high %EtOH (>80%) at low T_e_ (<35 °C), or with 20% EtOH at high T_e_ (>70 °C). To better understand these results, the polypeptide profile of alkaline extracts was studied by SDS-PAGE analysis under non-reducing conditions

#### 3.2.2. Effects of Optimal Aqueous Ethanol Extraction on Carinata Protein Profile

**Figure 4:**
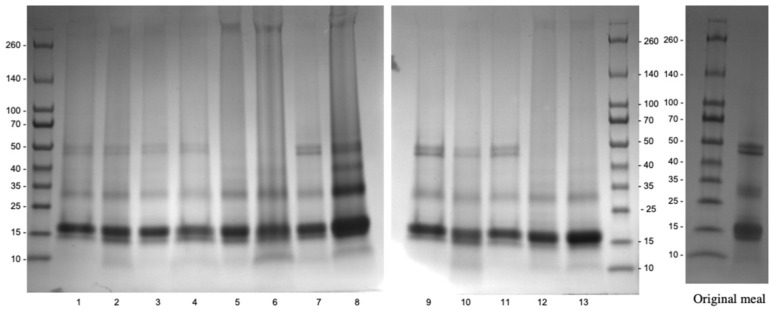
Polypeptide profiles of alkaline extracts from residual Carinata meal upon aqueous ethanol extraction. The numbers below indicate the experiments of D-optimal design described in Table 1.

Figure 4 presents the polypeptide profile obtained by SDS-PAGE for the different experiments of D-optimal design. The main Carinata proteins included, albumin (napin) and globulin (cruciferin), are attributed to the bands at 15 kDa and 50 kDa, respectively [6]. The bands at 30 kDa correspond to free polypeptide chains dissociated from cruciferin.

The most important difference between the profiles of alkaline extracts obtained during the experimental design and the control is the change of the 50 kDa bands (cruciferins). At an extraction temperature of 75 °C, the disappearance of these bands was observed (lanes 6, 12 and 13, Figure 4). Hence, cruciferin was either completely removed during the recovery of secondary metabolites, or dissociated when exposed to high temperatures during the AE extraction process [23].

The optimal operating conditions of the AE extraction process to maximize Y_3_, calculated by the MODDE software, are %EtOH = 90% and T_e_ = 25 °C. Under these conditions, the EI of proteins during alkaline extraction was the highest, and reached 76.0 ± 3.8%. No significant difference (Table S3) was found between the values predicted and those observed (78.8 ± 0.7%), thus validating the prediction model of EI.

Unfortunately, these optimal operating conditions are antagonistic to those for the recovery of secondary metabolites. Reaching a compromise leading to a high recovery of secondary metabolites while maintaining high protein EI is therefore discussed in the next section.

### 3.3. Determination of Operating Conditions for the Optimization of Y_1_, Y_2_, and Y_3_

**Figure 5:**
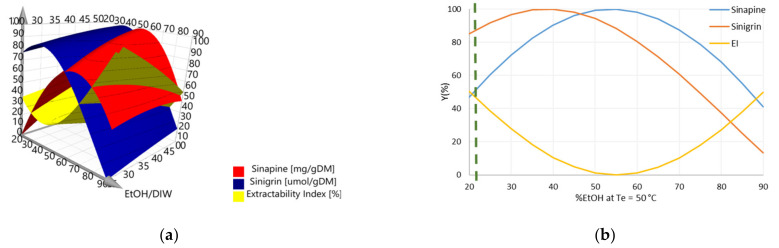
(**a**) Response surface plot of three main factors (PC content, GSL content, and EI) required to reach a compromise between the recovery of secondary metabolites and protein extractability upon AE treatment. The response values were scaled so that the minima and maxima were set at 0 and 100%, respectively; (**b**) Plotting chart to determine the desired compromise at 50 °C. The X axis was limited to 50% to visualize the reached compromise between selected responses.

A 3D graph representing the Y_1_, Y_2_, and Y_3_ responses as a function of *%EtOH* and T_e_ was drawn to visualize the areas for which the recovery of secondary metabolites is maximal, with minimal loss of protein extractability (Figure 5).

From the response surface plot generated by the experimental design software, it is difficult to define the precise operating conditions that would enable the best compromise between the three responses. This is due to complex antagonistic effects between the recovery of secondary metabolites and protein extractability, as depicted in Figure 5a. However, a more precise evaluation could be carried out using 2D graphs. Consequently, the three responses are plotted as a function of %EtOH, varying from 20 to 90% for a given extraction temperature. Figure 5b presents one of the graphs showing the evolution of Y_1_, Y_2_, and Y_3_ as a function of %EtOH, for T_e_ = 50 °C. To obtain the highest values for the three responses, 22% ethanol must be used. The other graphs are shown in Figure S3. For each graph, an optimal percentage of ethanol was determined. The results are presented in Table 3.

For each T_e_, the %EtOH resulting in the highest values of Y_1_, Y_2_, and Y_3_ was determined. The corresponding response values predicted by the model are also shown in Table 3.

Thus, it was noticed that the optimal %EtOH depends on T_e_. For temperatures below 40 °C, the optimal %EtOH is between 67 and 77%, whereas for temperatures of 45 and 50 °C, only 22 or 23% ethanol is needed to obtain the highest values of Y_1_, Y_2_, and Y_3_. These results are consistent with the two optimal zones described from Figure 3 for maximizing the protein extractability index.

According to Table 3, two combinations of operating conditions can be chosen to simultaneously reach the optimal values of the three responses. The first combination corresponds to a low T_e_ (25 °C) with 67% ethanol, which yields 9.94 ± 0.08 mg/g_DM_ of PC and 71.47 ± 0.90 µmol/ g_DM_ of GSL, with an IE of 62.15 ± 0.56%. These conditions maximize the PC content and the protein EI. The second combination allows an extraction at 50 °C with 22% EtOH to yield a PC content at 9.15 ± 0.09 mg/g_DM_, a GSL content at 91.02 ± 0.93 µmol/ g_DM_, and an EI of 61.12 ± 0.61%. In this case, it is the GSL content and the EI that are maximized. In order to develop a sustainable extraction process, our choice will be the second combination, which minimizes the amount of solvent used.

For these operating conditions, extractions were carried out, and experimental values were statistically equivalent to the values predicted by the models (Table S3). Thus, this reinforces the validation of the prediction models of Y_1_, Y_2_, and Y_3_, determined previously.

### 3.4. Effect of the New Operating Conditions on Functional Properties of Carinata Proteins upon Aqueous Ethanol Extraction

Functional properties of Brassica proteins, such as foaming and emulsifying capacities, are of great interest [10,40] and directly depend on their 3D structures [7]. According to the literature, these functional properties can be considered significant indicators of the effect of AE extraction on proteins. The emulsifying activity index (EAI), emulsifying stability (ES), foaming activity (FA), and foaming stability (FS) of protein isolates from original meal and treated meal under the selected extraction conditions (i.e., 22% EtOH at 50 °C) are shown in Table 4.

According to the Student’s *t*-test, EAI and FS values of protein isolates from both original and treated Carinata meal were quite similar. Although the ES of original meal was significantly higher than that of the treated meal, this loss remained low, considering the value of ES. The results were consistent with the functional properties of Carinata protein isolated at pH 12 reported by Pedroche et al. [3]. The ES value, on the other hand, corresponded to that of a protein isolate prepared at pH 10, as reported in the literature [3]. These differences might be due to the difference between the analytical methods employed.

The FA of treated meal was higher than that of original meal, whereas the FS of both protein isolates were similar, and were also comparable to protein isolate from defatted canola meal [8]. These results suggested that the removal of secondary metabolites modified the composition in proteins, and eventually their structure, leading to higher foaming capacity of protein isolate from treated meal.

Taken together, these results revealed that the changes in functional properties of treated meal upon AE extraction of Carinata meal with 22% EtOH at 50 °C improved the foaming activity, while preserving other functional properties of protein isolates.

## 4. Conclusions

Optimization of AE extraction of Carinata meal with aqueous ethanol resulted in validated models based on selected factors and responses. These models predicted that extraction with low %EtOH at moderate *T_e_* afforded the best balance between the efficiency of metabolite recovery from Carinata meal and the protein extractability of treated meals following AE extraction, while preserving the functional properties of protein isolates. Although metabolites recovered from Carinata meal were not individually separated in this study, preparative chromatography can be employed for this purpose. Moreover, the phytic acid recovery during the AE and alkaline extraction was disregarded in this study. Thereby, future work will address not only the separation of GSLs and PCs from AE extracts, but also the effects of our process on the recovery of phytic acid present in the meal. In addition, further research on Carinata proteins is ongoing, in order to determine the structural changes caused by AE extraction.

## Figures and Tables

**Table 1:** D-optimal experimental design.

Experiments	%EtOH (%) X_1_	T_e_ (°C) X_2_
0	0	25
1	70	50
2	20	25
3	70	50
4	20	50
5	45	50
6	20	75
7	70	25
8	70	50
9	90	25
10	20	25
11	90	50
12	45	75
13	70	75

**Table 2:** Model equation coefficients and statistical parameters.

Factors	Coefficient Values		
	Y_1_	Y_2_	Y_3_
Constant	1.0333	1.9744	−1.7016
%EtOH	0.0028 ^NS^	−0.0864	−0.0080 ^NS^
T_e_	0.0144 ^NS^	0.0221 ^NS^	−0.0529
%EtOH×%EtOH	−0.0457	−0.0847	0.0818
T_e_ × T_e_	−0.0145 ^NS^	−0.0126 ^NS^	−0.0108 ^NS^
%EtOH× T_e_	−0.0220	0.0143 ^NS^	−0.0717
R^2^	0.869	0.929	0.849
R^2^ adjusted	0.775	0.870	0.763
Regression (*p*-value)	5.4 × 10^−3^	2.2 × 10^−3^	5.3 × 10^−3^
Lack of fit	0.324	0.708	0.273
Reproducibility	0.847	0.820	0.900
Condition number	4.654	4.683	3.605

^NS^ denotes non-significant; coefficients in green are significant.

**Table 3:** Values of PC and GSL contents (*Y_1_* and *Y_2_*) and the extractability index (*Y_3_*) associated with each % EtOH optimum according to *T_e_*.

T_e_ (°C)	Optimal %EtOH	Y_1_ (mg/g_DM_)	Y_2_ (µmol/g_DM_)	Y_3_ (%)
25	67%	9.94 ^a^ ± 0.08	71.47 ^b^ ± 0.90	62.15 ^a^ ± 0.56
30	70%	10.10 ^a^ ± 0.08	70.31 ^b,c^ ± 0.92	61.56 ^a^ ± 0.57
35	73%	10.13 ^a^ ± 0.09	68.63 ^c,d^ ± 0.92	60.85 ^a,b^ ± 0.58
40	77%	10.01 ^a^ ± 0.09	66.89 ^d^ ± 0.93	60.45 ^b^ ± 0.59
45	23%	9.09 ^b^ ± 0.09	92.19 ^a^ ± 0.93	59.94 ^b^ ± 0.60
50	22%	9.15 ^b^ ± 0.09	91.02 ^a^ ± 0.93	61.12 ^a^ ± 0.61

Letters associated with the means correspond to equivalent values according to the Tukey’s test.

**Table 4:** Protein content and functional properties of Carinata protein isolates of original and treated meal.

Type of Meal	EAI (m^2^/g)	ES (%)	FA (%)	FS (%)
Original meal	10.7 ± 1.4	63.5 ± 0.2	123.8 ± 1.8	94.9 ± 1.5
Treated meal	10.0 ± 0.6	61.4 ± 1.1	147.3 ± 3.2	94.2 ± 1.6
Student’s test (*p*-value)	0.571	0.035	4.07E^−12^	0.467

## Data Availability

The data presented in this study are available in the article and in the supplementary file.

## Supplementary Materials

The following supporting information can be downloaded at: https://www.mdpi.com/article/10.3390/foods11030429/s1, Table S1. Phenolic compound content of AE and alkaline extracts; Table S2. Glucosinolate content of AE and alkaline extracts; Table S3. Extern validation of prediction models; Table S4. Extractability index (EI) of proteins of AE and alkaline extracts; Figure S1. Phenolic compound analysis by HPLC of AE extracts under different extraction conditions; Figure S2. Glucosinolates analysis by HPLC of AE extracts under different extraction conditions; and Figure S3. Plotting chart to determine desired compromise at different temperatures from 25 to 50 °C.
