# Supplementary material for: Optimization of the Recovery of Secondary Metabolites from Defatted Brassica carinata Meal and Its Effects on the Extractability and Functional Properties of Proteins
Source: Foods. 2022 Feb 1;11(3):429. doi: 10.3390/foods11030429 (PMC8834076; doi:10.3390/foods11030429)
Supplement: Supplementary file 1 [file foods-11-00429-s001.zip › foods-1575867-supplementary.pdf]

Supplementary Information

Optimization of the Recovery of Secondary Metabolites from defatted *Brassica carinata* meal and Its Effects on the Extractability and Functional Properties of Proteins

V. P. Thinh NGUYEN, [†,‡] *Jon D. STEWART,* [‡] *Florent ALLAIS*[*,†,‡] *and Irina IOANNOU*[*,†]

[†] URD ABI, CEBB, AgroParisTech, 51110 Pomacle, France

[‡] Department of Chemistry, University of Florida, 32611 Gainesville, Florida, USA

Experimental data obtained for the D-optimal design are presented in **Table S1** for Y_1 (Phenolic compound content) and in **Table S2** for Y_2 (Glucosinolate content).

Table S1. Phenolic compound content of AE and alkaline extracts.

Entries	%EtOH	T_e (°C)	Phenolic compound content of AE extracts (mg/g_{DM})	Phenolic compound content of alkaline extracts (mg/g_{DM})
0	0	25	-	6.6723
1	70	50	10.3764	1.4336
2	20	25	8.0726	1.4033
3	70	50	9.8962	1.2599
4	20	50	8.6998	1.2901
5	45	50	11.3354	1.1287
6	20	75	10.0643	1.2110
7	70	25	10.4755	1.6094
8	70	50	10.3432	1.4518
9	90	25	8.6687	1.9641
10	20	25	7.2657	1.6398
11	90	50	9.4402	1.8860
12	45	75	10.2762	1.3258
13	70	75	9.7151	1.4688

The model predicting the phenolic compound content with unscaled coefficients is shown in **Equation S1**:

$$\text{Log}(Y_1) = 0.6028 + 0.0090\%\,EtOH + 0.0069\,Te - (6.3289 \times 10^{-5})\%ETOH^2$$
$$-(4.0226{\times}10^{-5})T_e^2 - (4.3260 \times 10^{-5})\%EtOHT_e, \quad \text{(S1)}$$

Table S2. Glucosinolate content of AE and alkaline extracts.

Entries	%EtOH	T_e (°C)	Glucosinolate content of AE extracts (µmol/g_{DM})	Glucosinolate content of alkaline extracts (µmol/g_{DM})
0	0	25	-	70.7482
1	70	50	72.0563	13.3285
2	20	25	88.1575	7.6720
3	70	50	87.7529	12.3955
4	20	50	92.3045	12.6897
5	45	50	93.6177	2.0007
6	20	75	94.0140	-0.0395
7	70	25	74.3477	25.5384
8	70	50	32.5855	14.9310
9	90	25	42.5303	42.7234
10	20	25	79.1531	14.9071

11	90	50	47.6201	41.1014
12	45	75	91.6635	5.0773
13	70	75	83.1287	6.3332

The model predicting the glucosinolate content with unscaled coefficients is shown in **Equation S2**:

$$\mathrm{Log}(Y_2) = 1.7708 + 0.0073\%\,EtOH + 0.0028\,Te - (1.1128 \times 10^{-4})\%ETOH^2 \\ -(3.2136 \times 10^{-5})T_e^2 - (2.60154 \times 10^{-5})\%EtOHT_e, \quad \text{(S2)}$$

An extern validation of the models generated by the D-optimal design was carried out for the two optimal operating conditions found in the study. Results are presented **Table S3**.

Table S3. Extern validation of prediction models.

	Y_1 (mg/g)	Y_2 (mg/g)	Y_3 (%)
	**Conditions optimizing Y_1 and Y_2 (47% ethanol, 62 °C)**		**Conditions optimizing Y_3 (90% ethanol, 25 °C)**
Predicted values	10.87 ± 0.54	98.96 ± 4.9	76 ± 3.8
Observed values	11.74 ± 1.26	103.6 ± 16.2	78.8 ± 0.7
p-value (Student test)	0.132	0.722	0.544
**Condition optimizing Y_1, Y_2 and Y_3 (22% ethanol, 50 °C)**			
Predicted values	9.15 ± 0.09	91.02 ± 0.93	61.12 ± 0.61
Observed values	9.12 ± 0.05	86.54 ± 3.18	59.8 ± 2.1
p-value (Student test)	0.64	0.08	0.35

Experimental data obtained for the D-optimal design are presented in **Table S4** for Y_3 (extractability index of proteins)

Table S4. Extractability index (EI) of proteins of AE and alkaline extracts.

Entries	%EtOH	T_e (°C)	Protein EI of AE extracts (%)	Protein EI of alkaline extracts (%)
0	0	25	-	59.22
1	70	50	41.3	64.26
2	20	25	48.5	65.75
3	70	50	42.5	64.9
4	20	50	46.5	67.3
5	45	50	48.7	63.8
6	20	75	47.2	58.7
7	70	25	42.3	67.2
8	70	50	46.9	62.7
9	90	25	44.4	63.6
10	20	25	48.3	67.3
11	90	50	44.5	63.8
12	45	75	54.5	58.1
13	70	75	51.3	55.7

The model predicting the extractability index with unscaled coefficients is shown in **Equation S3**:

$$-\mathrm{Log}(100\text{-}Y_3) = -1.5924 - 0.0053\%\,EtOH + 0.0042\,Te - (1.0742 \times 10^{-4})\%ETOH^2 - (1.3123 \times 10^{-4})\%EtOHT_e, \quad \text{(S3)}$$

The analysis by HPLC of Carinata meal's aqueous ethanol extraction was conducted and shown in **Figure S1**. The variation of extractions conditions resulted in different sinapine concentrations extracted sinapine concentrations.

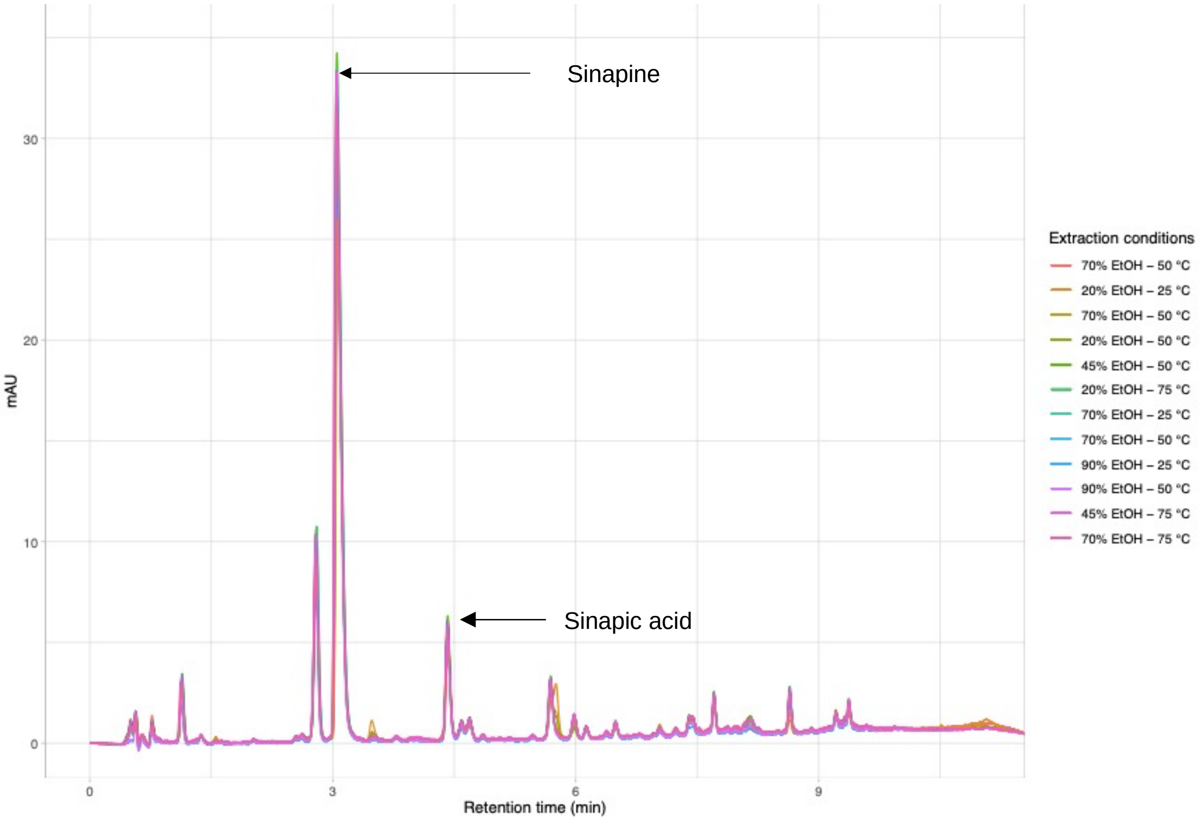

Figure S1. Phenolic compounds analysis by HPLC of aqueous ethanol extracts under different extraction conditions. Samples were prepared and analyzed by HPLC as described in our previous study [22]. Chromatograms were recorded at 320 nm.

The analysis of aqueous ethanol extraction effect on Carinata meal's glucosinolates was conducted and shown in **Figure S2**. The variation of extractions conditions resulted in different extracted desulfated sinigrin concentrations.

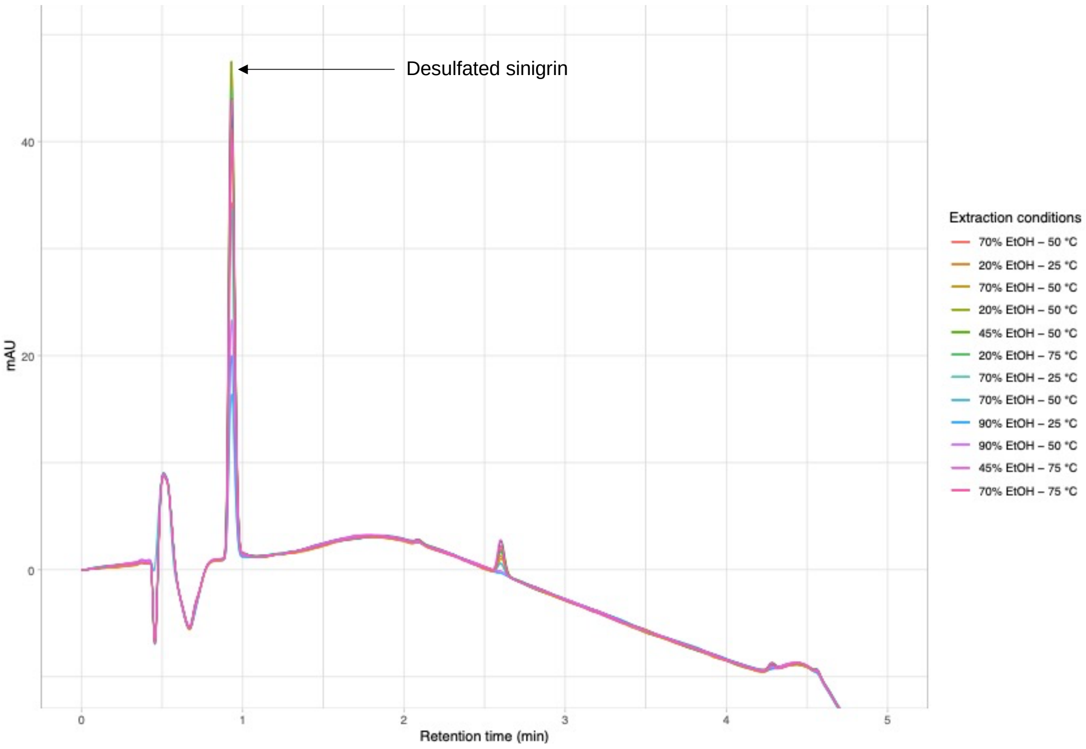

Figure S2. Glucosinolates analysis of aqueous ethanol extract at different extraction conditions. Samples were prepared and analyzed by HPLC as described by Grosser and van Dam [32]. Chromatograms were recorded at 229 nm.

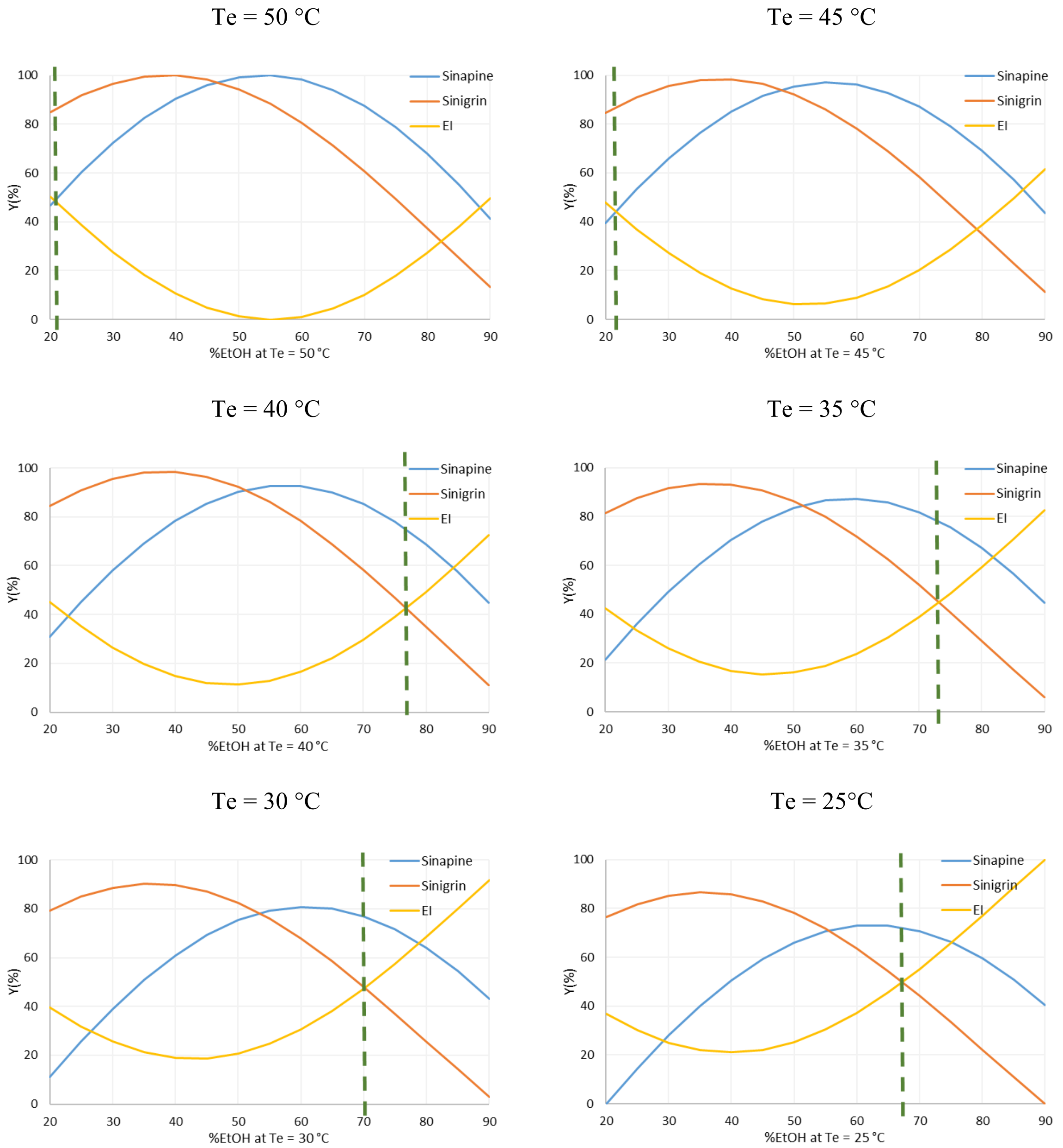

Figure S3. Plotting chart to determine desired compromise at different temperatures from 25 to 50 °C. The response values were scaled where the minima and the maxima were set at 0 and 100%, respectively. The X axis presents %EtOH; and Y axis presents scaled responses.
